# Supplementary material for: Cytosolic sorting platform complexes shuttle type III secretion system effectors to the injectisome in Yersinia enterocolitica
Source: Nat Microbiol. 2024 Jan 3;9(1):185–99. doi: 10.1038/s41564-023-01545-1 (PMC10769875; doi:10.1038/s41564-023-01545-1)
Supplement: Supplementary file 1 — Supplementary Figs. 1–11, Tables 1–6 and Text 1–6. [file 41564_2023_1545_MOESM1_ESM.pdf]

nature microbiology

Article

https://doi.org/10.1038/s41564-023-01545-1

Cytosolic sorting platform complexes shuttle type III secretion system effectors to the injectisome in *Yersinia enterocolitica*

In the format provided by the authors and unedited

Table of contents

Suppl. Fig. 1: Stability and functionality of N-terminal PAmCherry fusions to sorting platform components

Suppl. Fig. 2: Documentation of protein purification in co-immunoprecipitation experiments.

Suppl. Fig. 3: Expression and stability of T3SS components in *Y. enterocolitica* lacking the pYV virulence plasmid

Suppl. Fig. 4: Free diffusion of PAmCherry in *Y. enterocolitica* pYV$^-$

Suppl. Fig. 5: The sorting platform proteins do not display a high turnover in live bacteria

Suppl. Fig. 6: Quantification of PAmCherry-SctQ foci per bacterial cell

Suppl. Fig. 7: Quantitative analysis of PAmCherry trajectories per injectisome

Suppl. Fig. 8: T3SS effector YopH and chaperone SycH are localized in the cytosol

Suppl. Fig. 9: Quantification of sptPALM data and spatial filtering for cytosolic fraction of SctQ diffusion

Suppl. Fig. 10: Free diffusion of PAmCherry in wild-type *Y. enterocolitica*

Suppl. Fig. 11: Protein stability and overall biotinylation levels in proximity labeling experiments

Suppl. Table 1: Label-free mass spectrometry quantification of proximity labeling of T3SS-associated proteins

Suppl. Table 2: Number of measured trajectories and replications in single particle tracking experiments in this study

Suppl. Table 3: Label-free mass spectrometry quantification of cellular amounts of the indicated T3SS substrates and machinery components.

Suppl. Table 4: List of plasmids and strains used in this study

Suppl. Table 5: List of oligonucleotides used in this study

Suppl. Table 6: Assignment of raw data of proteomics files

Suppl. Text 1: Predicting diffusion behavior of the T3SS sub-complexes

Suppl. Text 2: Variety of T3SS sub-complexes

Suppl. Text 3: Detection rate of PAmCherry-SctQ molecules and quantification of injectisomes per bacterium

Suppl. Text 4: Jump distances as a measure of particle movement

Suppl. Text 5: Composition and stoichiometry of T3SS sorting platform pods

Suppl. Text 6: Calculation of SctQ exchange rate at single injectisomes

Legends for:

Suppl. Video 1-4: Examplary raw microscopy data of single particle tracking photoactivated localization microscopy

Suppl. Data 1: Raw data and statistical analysis of sptPALM measurements in *Y. enterocolitica*

Suppl. Data 2: Source Data files for Supplementary Figures

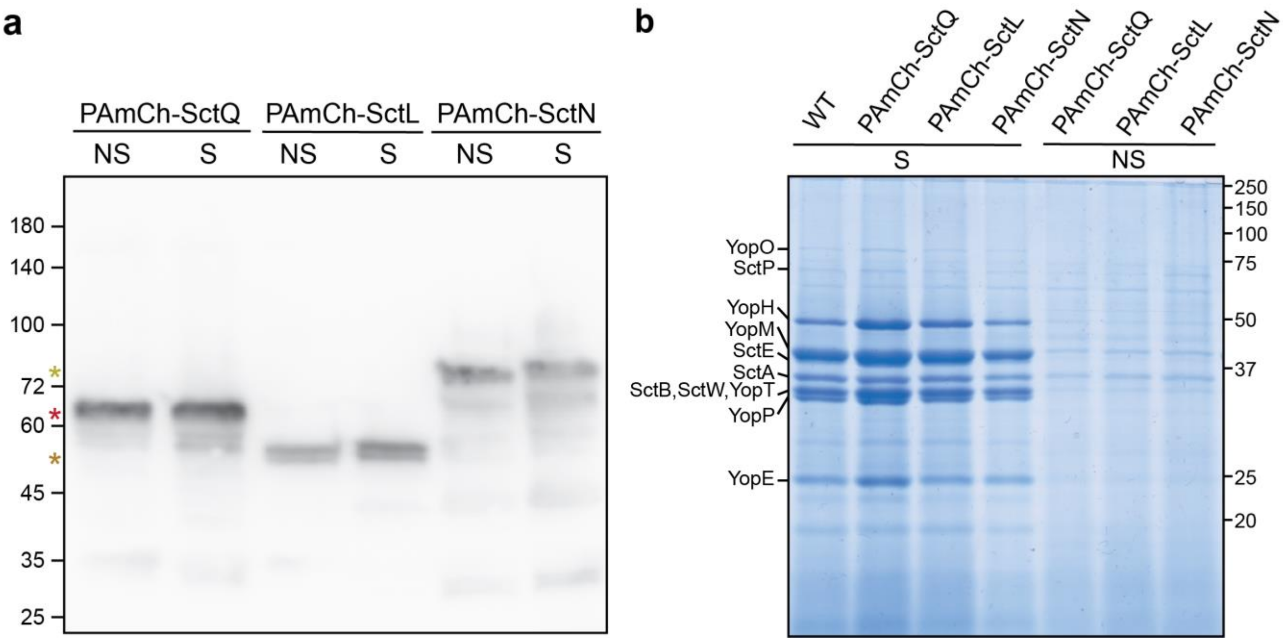

Suppl. Fig. 1: Stability and functionality of N-terminal PAmCherry fusions to sorting platform components

a) Immunoblot with anti-mCherry antibody detecting the indicated PAmCherry (PAmCh) fusion proteins, expressed from their native genetic background under non-secreting (NS) and secreting (S) conditions. Asterisks indicated the expected monomeric molecular weights of PAmCherry-SctQ (red, 62.5 kDa), PAmCherry-SctL (orange, 53.6 kDa), and PAmCherry-SctN (yellow, 77.3 kDa). Left, molecular weight marker in kDa. **b**) Secretion assay of strains expressing the indicated fusion proteins. Precipitated proteins from culture supernatant, bands indicate exported effector proteins (names on left side). Right, molecular weight marker in kDa. *n*=3.

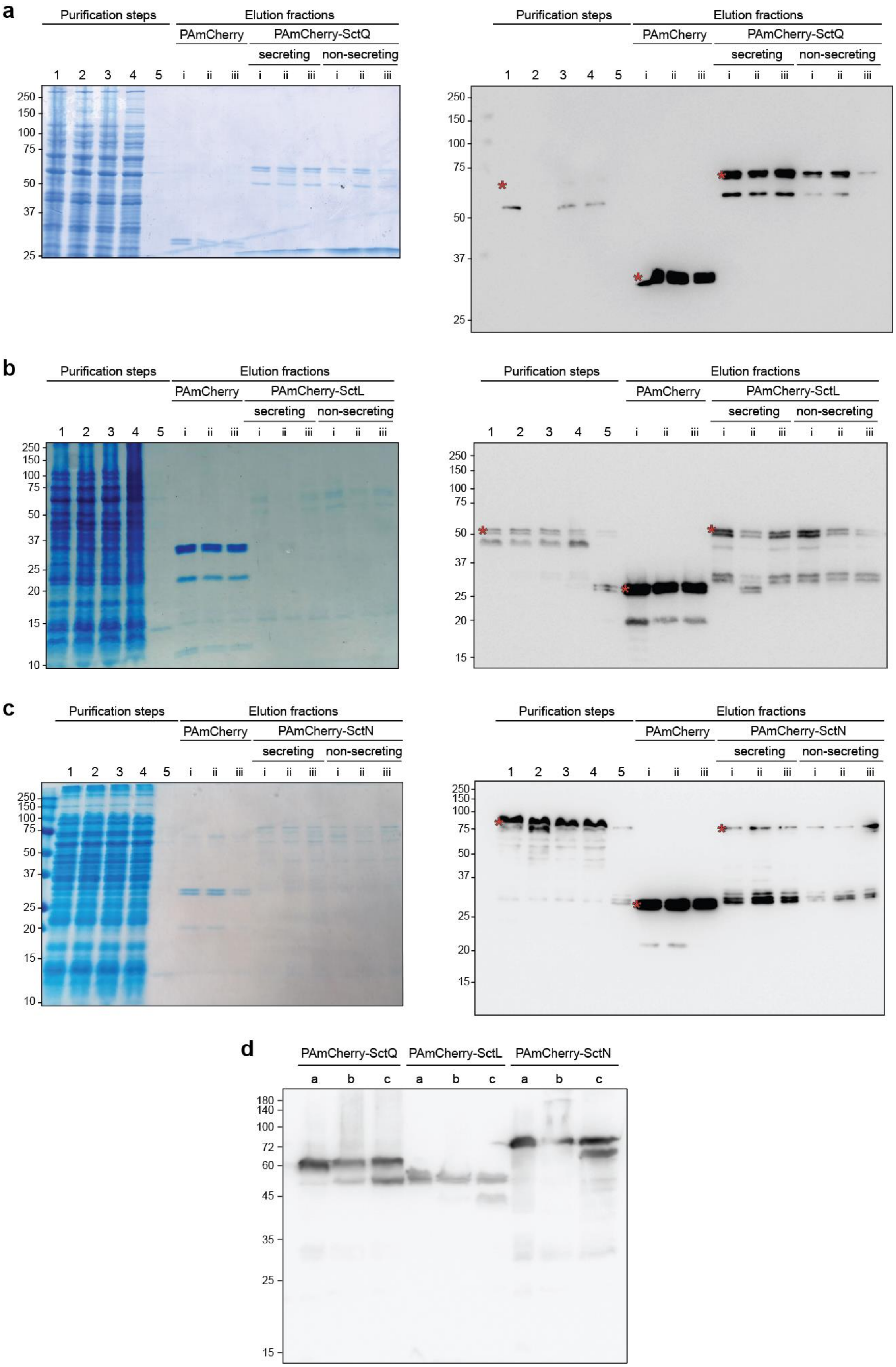

Suppl. Fig. 2: Documentation of protein purification in co-immunoprecipitation experiments.

a) - **c**), Indicated fractions of the co-immunoprecipitation of strains expressing PAmCherry-SctQ (**a**), PAmCherry-SctL (**b**), or PAmCherry-SctN (**c**) were separated on 11% SDS-PAGE gels and visualized with instant blue staining (left) and immunoblots anti-mCherry (right). Left, molecular weight marker (size in kDa). Purification steps shown for respective fusion proteins under secreting conditions: 1) Total cellular protein prior to lysis; 2) after lysis by French press; 3) after removal of insoluble fraction by centrifugation; 4) supernatant after incubation with purification beads; 5) last wash fraction from beads before elution. Elution fractions shown for three biological replicates (i-iii) of the indicated strains and conditions. Asterisks mark proteins of interest (exp. monomeric mol. weight: PAmCherry-SctQ = 62.5 kDa, PAmCherry-SctL= 53.6 kDa, PAmCherry-SctN = 77.3 kDa). **d**) Stability of the fusion proteins under the following conditions relevant for the purification process: a, directly after incubation at 37°C and prior to wash and resuspension in lysis buffer; b, after resuspension and incubation with HNN lysis buffer; c, after thawing, directly prior to lysis. See methods for details. *n*=3. Left, molecular weight marker (size in kDa).

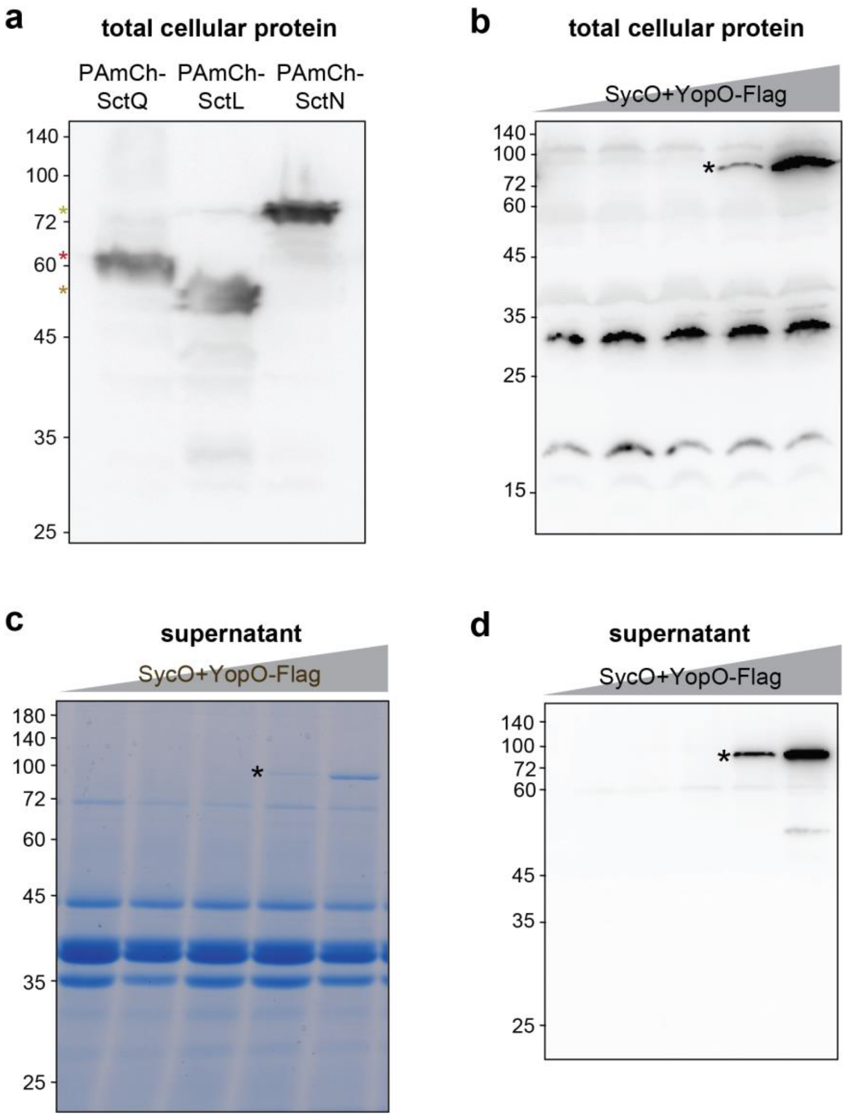

Suppl. Fig. 3: Expression and stability of T3SS components in *Y. enterocolitica* lacking the pYV virulence plasmid

a) Immunoblot anti-mCherry on total cellular protein samples from *Y. enterocolitica* expressing the indicated fusion proteins in a strain lacking the virulence plasmid (pYV$^-$). Asterisks indicated the expected monomeric molecular weights of PAmCherry-SctQ (red, 62.5 kDa), PAmCherry-SctL (orange, 53.6 kDa), and PAmCherry-SctN (yellow, 77.3 kDa). **b**) Immunoblot anti-Flag on total cellular protein samples from *Y. enterocolitica* expressing SycO and YopO-Flag from plasmid in a wild-type Δeffector strain. Triangle indicates increasing expression levels of SycO/YopO-Flag (induction with no, 0.002%, 0.01%, 0.05%, 0.25% arabinose, respectively). **c)** Secretion assay of strains used in b) showing secretion of YopO-Flag. **d**) Immunoblot anti-Flag of samples used in c). Asterisks mark the expected molecular weight of YopO-Flag = 82.7 kDa. *n*=3.

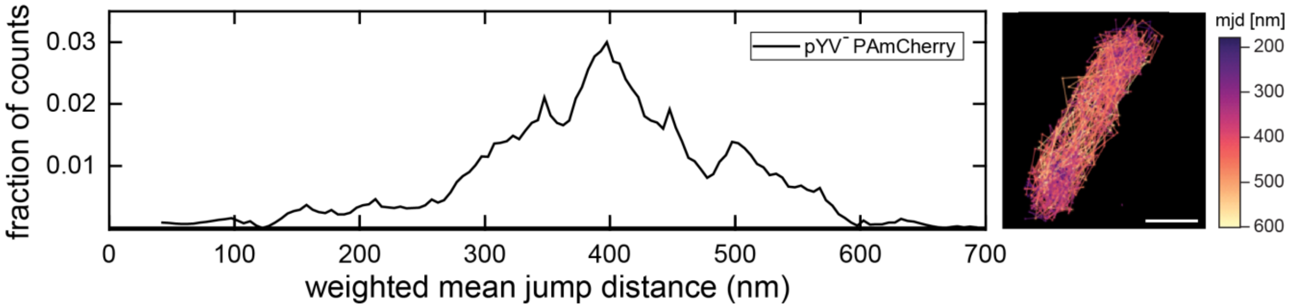

Suppl. Fig. 4: Free diffusion of PAmCherry in *Y. enterocolitica* pYV^{-}

Left, mobility of PAmCherry expressed from plasmid in *Y. enterocolitica* lacking all other components of the T3SS (pYV^{-}). Histogram of mean jump distances (mjd) of molecular diffusion, weighted for the number of jump distances. Right, trajectories in representative bacterium. Scale bar, 0.5 µm. Numbers of trajectories and replications for single particle tracking experiments are summarized in Suppl. Table 2.

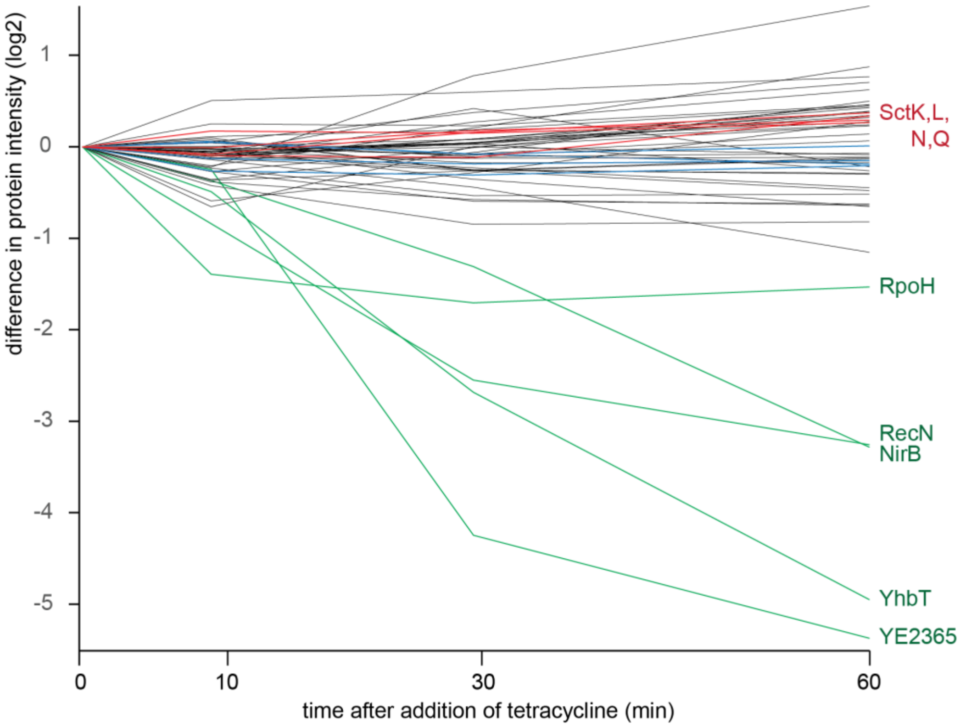

Suppl. Fig. 5: The sorting platform proteins do not display a high turnover in live bacteria

Changes in protein levels of cellular proteins of wild-type *Y. enterocolitica* before and after inhibition of new protein synthesis by addition of tetracycline, quantified by label-free mass spectrometry. The sorting platform proteins marked by red lines (from top to bottom at 60 min: SctK, SctL, SctN, SctQ), are not significantly degraded in the measured time period, similar to the other detected T3SS components (black lines). Selected stable proteins (from top to bottom at 60 min: 30S ribosomal protein S7, chaperone protein DnaK, chromosome partition protein MukF, membrane protein HemY) are marked in blue; selected proteins with high turnover [1–3] are marked in green and indicated on the right side. *n*=2.

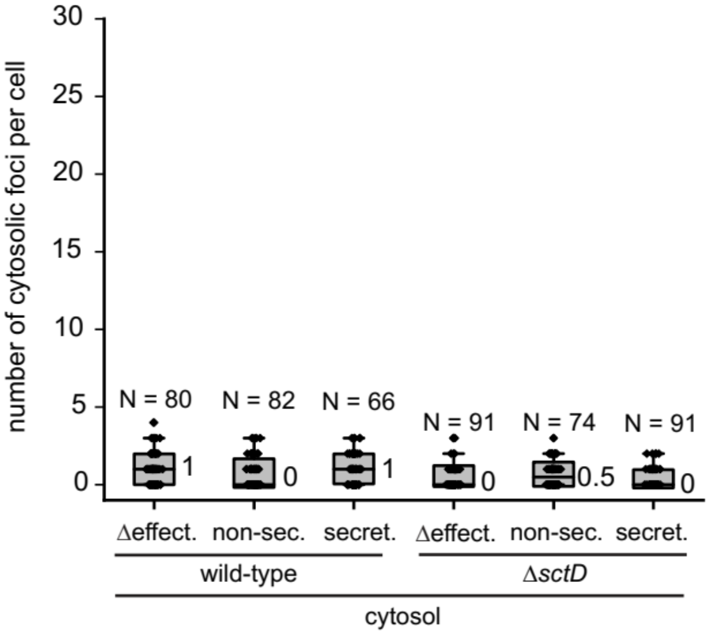

Suppl. Fig. 6: Quantification of PAmCherry-SctQ foci per bacterial cell

In contrast to the distinct injectisome foci at the membrane (see Fig. 2bc), bacteria have very few or no foci in the cytosol. Quantification of membrane-bound stable PAmCherry-SctQ foci per bacterial cell detected by sptPALM in the indicated *Y. enterocolitica* strain backgrounds and conditions. Numbers next to each bar plot indicate the corresponding median value, total numbers of analyzed bacterial cells are indicated on top of each bar plot. Box denotes mean and standard deviation; whisker range corresponds to 5-95%. **b**) Quantification of cytosolic stable PAmCherry-SctQ foci per bacterial cell detected by sptPALM in the indicated *Y. enterocolitica* strain backgrounds and conditions. Numbers next to each bar plot indicate the corresponding median value, total numbers of analyzed bacterial cells are indicated on top of each bar plot. Box denotes mean and standard deviation; whisker range corresponds to 5-95%.

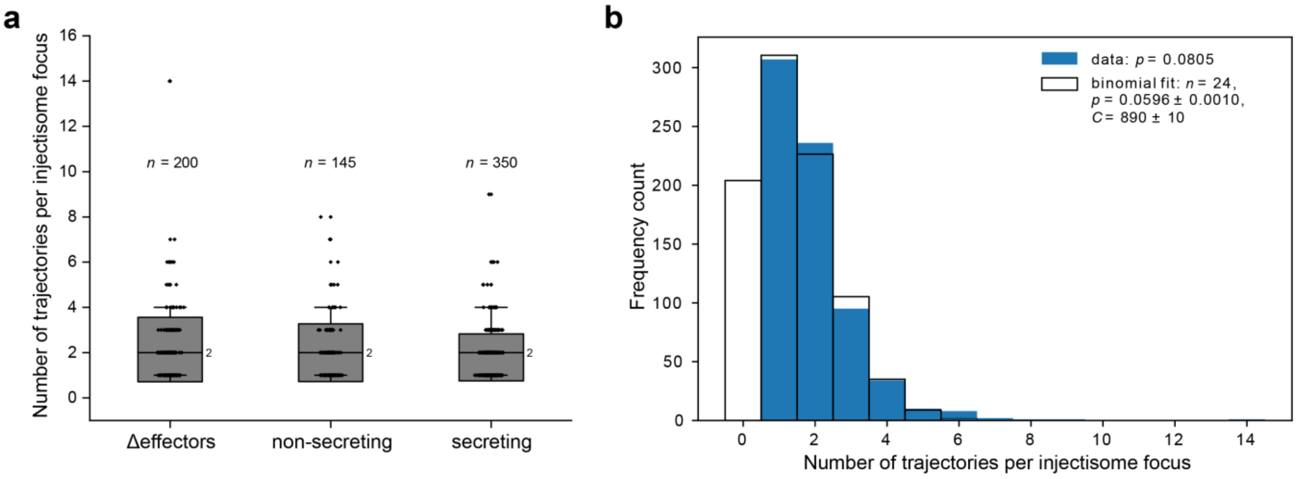

Suppl. Fig. 7: Quantitative analysis of PAmCherry trajectories per injectisome

a) Quantification of PAmCherry trajectories per stable injectisome (see Materials and Methods for details) detected by sptPALM in the indicated *Y. enterocolitica* strain backgrounds and conditions. Numbers next to each bar plot indicate the corresponding median value, total numbers of analyzed injectisomes are indicated on top of each bar plot. Box range corresponds to the standard deviation, whisker range to 5-95% of all data points. **b**) Binomial distribution fit analysis (see Materials and Methods for details) of combined histogram data shown in a) with a bin size of 1, with the blue histogram representing measured data, and the transparent bordered histogram representing the binomial fit. Parameters fitted: *p,* detection probability; C, sum of all detected PAmCherry molecules (shown in a) and not detected)).

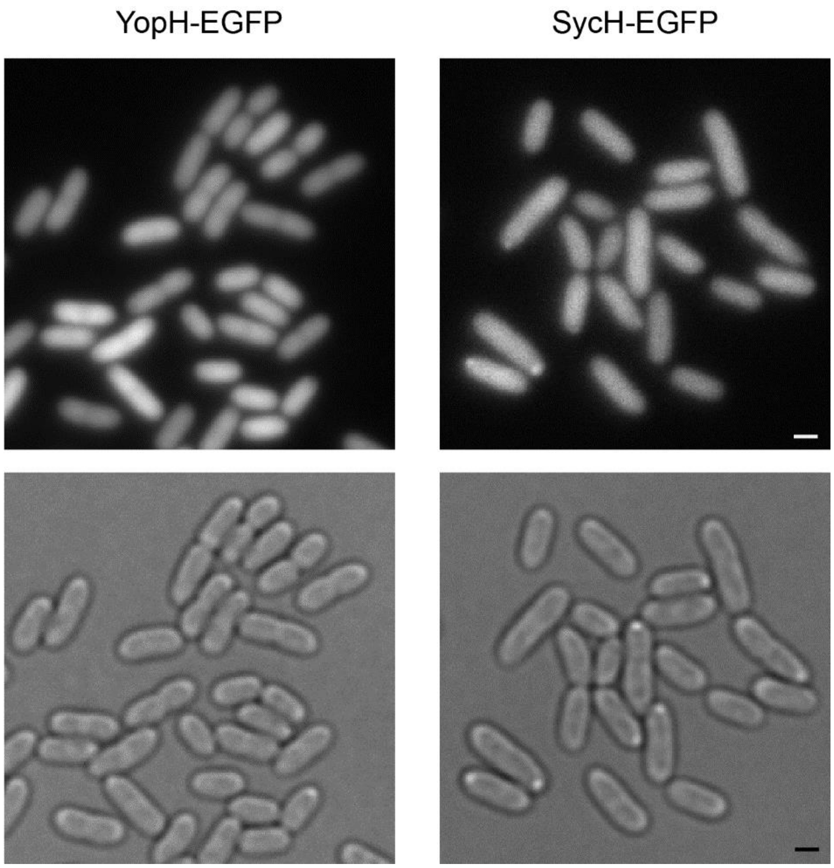

Suppl. Fig. 8: T3SS effector YopH and chaperone SycH are localized in the cytosol

Micrographs of strains expressing the indicated fusion proteins from their native genetic background under secreting conditions. Top, GFP fluorescence micrographs (intensity scaled 1:8 for YopH-EGFP); bottom, respective differential interference contrast (DIC) micrographs. Scale bars, 1 µm. Corresponding partial / complete cytosolic localization of EGFP-SctQ and EGFP-SctL (in presence / absence of fully assembled injectisomes, respectively) in references [4] and [5], corresponding sptPALM data in Fig. 1c, 2b, 3. *n* = 3.

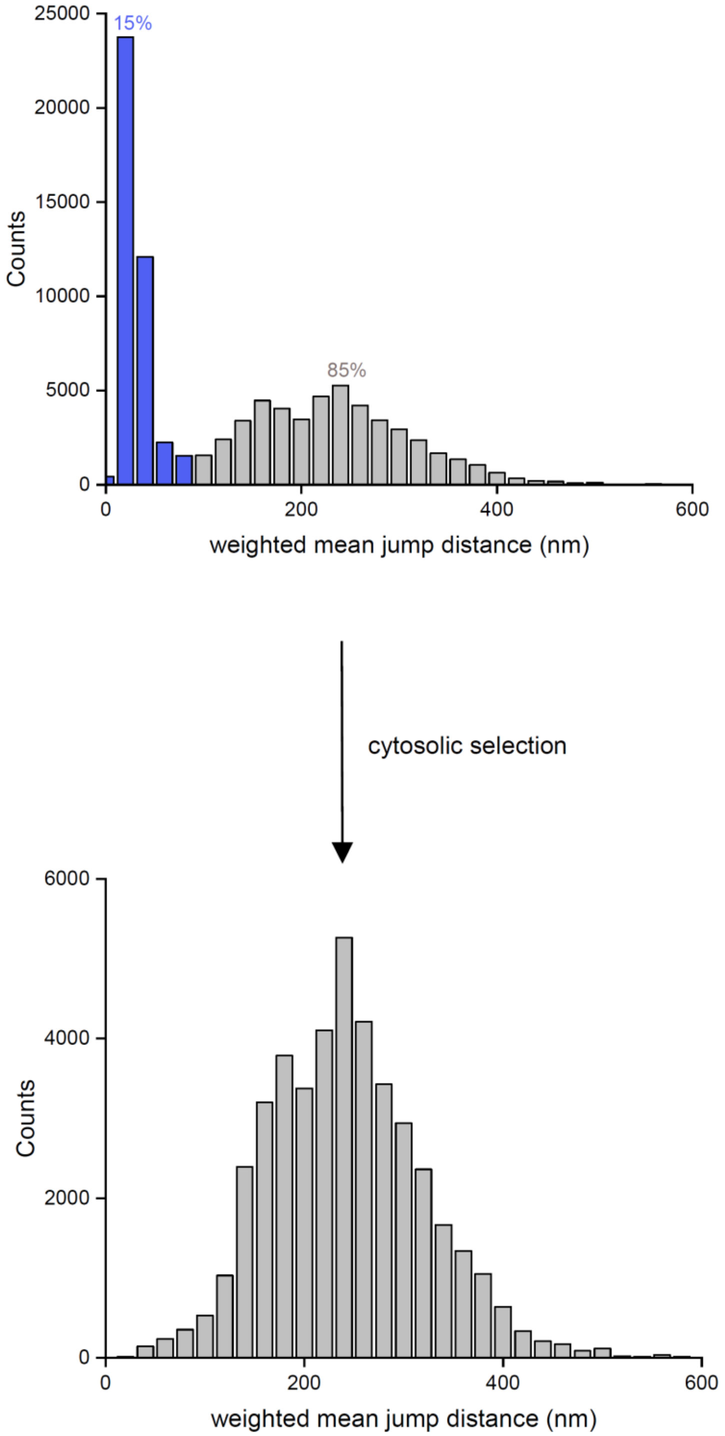

Suppl. Fig. 9: Quantification of sptPALM data and spatial filtering for cytosolic fraction of SctQ diffusion

Histograms displaying the mean jump distances (MJD) of PAmCh-SctQ of *Y. enterocolitica* wild-type cells. Top diagram includes all tracks; 85% of SctQ molecules are diffusive, whereas 15% appear as immobile SctQ foci. Bottom diagram only includes cytosolic tracks, showing that immobile SctQ foci appear exclusively at the membrane.

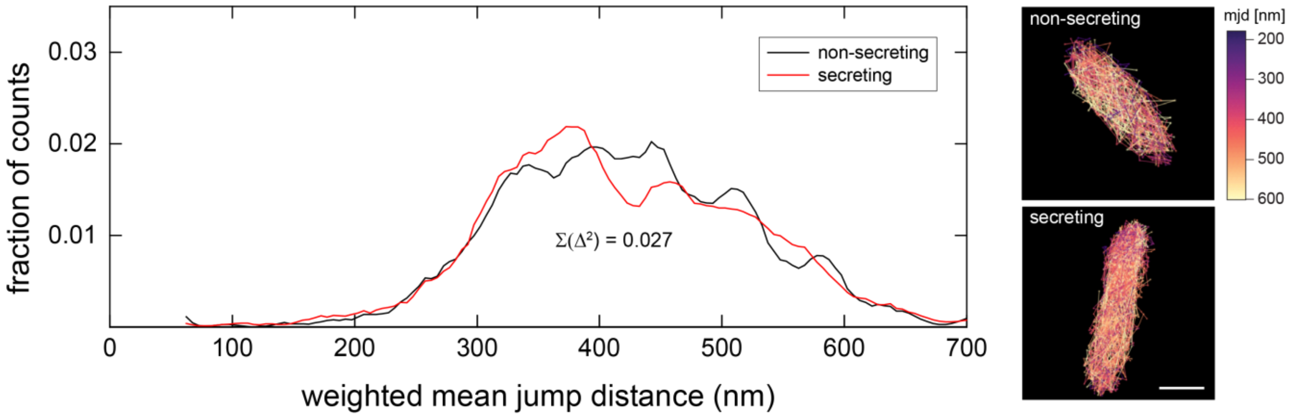

Suppl. Fig. 10: Free diffusion of PAmCherry in wild-type *Y. enterocolitica*

Left, mobility of PAmCherry expressed from plasmid in *Y. enterocolitica* wild-type bacteria. Histogram of mean jump distances (mjd) of trajectories, weighted for the number of jump distances. The mobility is similar under secreting and non-secreting conditions ($\Sigma(\Delta^2) = 0.027$; $r^2 = 0.941$). Right, trajectories in representative bacterium. Scale bar, 0.5 µm. Numbers of trajectories and replications for single particle tracking experiments are summarized in Suppl. Table 2.

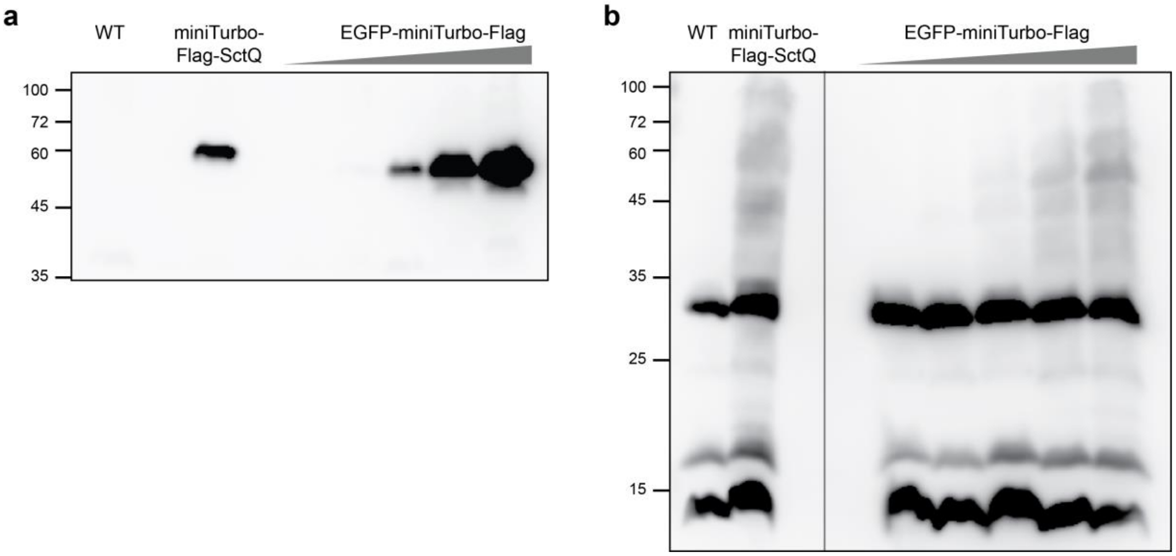

Suppl. Fig. 11: Protein stability and overall biotinylation levels in proximity labeling experiments

a) Immunoblot anti-Flag of cellular proteins of *Y. enterocolitica* wild-type (WT) and strains expressing miniTurbo-Flag-SctQ from its native genetic location or EGFP-miniTurbo-Flag from plasmid with increasing induction levels (left to right: arabinose concentrations of 0%, 0.01%, 0.03%, 0.1%, 0.3%). Based on these results, an arabinose concentration of 0.1% was used for the subsequent experiments. Left, molecular weight marker in kDa; expected molecular weight: miniTurbo-Flag-SctQ, 65.0 kDa; EGFP-miniTurbo-Flag, 58.1 kDa. **b**) Immunoblot using streptavidin-HRP conjugate to detect biotinylated proteins in samples used in a). *n*=3 biologically independent samples for both subpanels.

Suppl. Table 1: Label-free mass spectrometry quantification of proximity labeling of T3SS-associated proteins

Log_2 intensity values for all proteins encoded on the pYV virulence plasmid with at least three detected peptides. Strains as indicated, EGFP-miniTurbo served as a control for unspecific biotinylation by cytosolic miniTurbo protein expressed at a level comparable to the natively expressed miniTurbo-SctQ. Eluate = Proteins detected after streptavidin-based purification of biotinylated proteins. Lysate = Proteins detected in cell lysate prior to purification (expression control). Three biological replicates (two for lysate of untagged wild-type strain), as indicated. Blue, sorting platform proteins; red, exported T3SS effectors; orange, exported T3SS translocators. Effectors (red) are consistently enriched, whereas this is not the case for the translocators (orange). See Material and Methods for details.

Protein	Function	Eluate								Lysate										
		EGFP-miniTurbo			miniTurbo-SctQ			EGFP-mini Turbo	mini Turbo -SctQ	untagged wild-type (WT)		EGFP-miniTurbo			miniTurbo-SctQ			WT	EGFP-mini Turbo	mini Turbo -SctQ
		Repl. 1	Repl. 2	Repl. 3	Repl. 1	Repl. 2	Repl. 3	Avg.	Avg	Repl. 1	Repl. 2	Repl. 1	Repl. 2	Repl. 3	Repl. 1	Repl. 2	Repl. 3	Avg	Avg	Avg
SctQ	Sorting platform (miniTurbo-labeled bait protein)	30.62	30.50	30.56	34.60	34.68	34.57	30.56	34.62	33.91	33.36	33.58	33.52	33.55	33.75	33.92	33.70	33.66	33.55	33.79
YopH	Exported effector protein	32.31	32.21	32.73	34.18	32.83	33.78	32.43	33.70	35.01	36.35	35.87	35.28	35.24	35.91	35.04	35.85	35.83	35.49	35.65
YopT	Exported effector protein	26.03	25.89	26.27	27.62	26.83	27.11	26.07	27.22	29.56	30.86	30.08	29.58	29.68	30.38	29.50	30.13	30.35	29.79	30.05
YopQ	Exported effector protein	27.93	28.06	28.25	29.66	28.62	28.79	28.09	29.10	32.30	33.76	33.28	32.72	32.76	33.39	32.60	33.20	33.21	32.94	33.10
SctL	Sorting platform	26.98	26.99	27.29	28.34	27.79	27.95	27.10	28.04	32.34	32.20	32.41	32.24	32.21	32.27	32.22	32.23	32.27	32.29	32.24
SctW/YopN	Exported gatekeeper protein	27.24	27.38	27.65	28.86	27.58	28.27	27.43	28.33	33.62	33.26	33.80	33.31	32.95	33.90	33.63	33.40	33.45	33.40	33.65
YopM	Exported effector protein	30.14	30.29	30.33	31.50	30.46	30.79	30.26	30.99	34.80	34.81	34.59	34.36	34.31	34.61	34.41	34.60	34.81	34.42	34.54
YopP	Exported effector protein	29.04	28.82	28.93	30.11	28.92	29.60	28.93	29.62	32.26	33.75	33.29	32.83	32.82	33.34	32.51	33.19	33.19	33.00	33.06
YscH/YopR	Exported effector protein	29.22	29.25	29.30	30.27	29.80	29.57	29.26	29.91	31.77	31.21	32.11	31.72	31.40	31.84	31.75	31.58	31.52	31.77	31.73
SctV	Export apparatus	25.93	25.59	26.07	26.70	26.37	26.26	25.87	26.46	31.13	30.95	31.17	30.95	31.12	30.99	31.06	30.84	31.04	31.08	30.96
YscX	Exported protein	25.80	25.70	25.99	26.76	26.10	26.23	25.83	26.39	30.98	31.56	31.56	31.26	31.10	31.51	31.23	31.27	31.30	31.32	31.34
SctE	Hydrophobic translocator / pore	30.72	30.57	31.00	31.73	30.50	30.90	30.78	31.14	35.54	35.85	36.07	35.63	35.68	35.89	35.50	35.55	35.70	35.81	35.65
YopQ	Exported effector protein	28.91	28.94	29.00	28.84	29.01	29.70	28.95	29.23	32.95	33.85	33.63	33.15	33.17	33.77	33.11	33.42	33.47	33.33	33.46
SctN	ATPase	26.76	26.86	26.76	27.29	26.95	26.95	26.80	27.07	31.08	31.10	31.21	31.16	31.26	31.23	31.23	31.25	31.09	31.21	31.24
SycH	Chaperone	25.24	25.54	25.07	25.68	25.52	25.12	25.29	25.46	31.42	31.79	31.69	31.40	31.62	32.01	31.66	31.79	31.62	31.57	31.82
YopE	Exported effector protein	31.97	31.79	32.17	32.53	31.21	32.05	31.98	32.03	36.62	36.47	36.50	36.26	36.38	36.55	36.30	36.29	36.55	36.38	36.38
SctJ	Inner membrane ring protein	28.38	28.16	28.45	28.61	28.19	28.22	28.34	28.35	32.42	32.44	32.58	32.35	32.36	32.56	32.48	32.54	32.43	32.43	32.53
YscG	Chaperone	30.70	30.97	30.69	30.65	30.73	30.91	30.79	30.77	33.29	32.99	33.12	32.81	33.10	33.22	33.32	33.00	33.14	33.02	33.19
SctC	Outer membrane ring protein / secretin	27.32	26.99	27.28	27.46	27.03	26.98	27.20	27.17	32.35	32.46	32.49	32.47	32.63	32.50	32.47	32.49	32.41	32.53	32.48
SctB	Hydrophobic translocator / pore	34.67	34.48	34.77	34.75	34.35	34.62	34.64	34.58	36.48	36.39	36.51	36.32	36.29	36.42	36.12	36.37	36.44	36.38	36.31
YscE	Chaperone	29.15	29.15	28.80	29.46	28.52	28.70	29.04	28.96	33.41	33.51	33.47	33.64	33.65	33.58	33.61	33.68	33.46	33.59	33.62
SctF	Needle subunit	30.93	30.87	31.24	31.27	30.34	30.78	31.02	30.85	34.01	34.08	34.34	33.84	34.09	34.15	34.27	33.92	34.04	34.10	34.12
SpyA	Virulence plasmid partitioning	26.77	26.88	26.60	26.85	26.42	26.33	26.75	26.55	30.05	30.27	30.48	30.49	30.28	30.39	30.19	30.41	30.16	30.42	30.33
SctA	Hydrophilic translocator / tip	32.47	32.38	32.34	32.24	32.18	32.17	32.40	32.20	34.01	34.10	34.27	33.90	34.11	34.35	34.32	34.20	34.06	34.10	34.29

SpyB	Virulence plasmid partitioning	27.08	26.84	27.21	27.13	26.54	26.79	27.05	26.84	30.28	30.21	30.19	30.12	30.16	30.10	30.19	30.25	30.25	30.16	30.18
SycD	Chaperone	29.66	29.49	29.50	29.47	29.23	29.27	29.55	29.33	34.64	34.35	34.51	34.16	34.28	34.53	34.34	34.19	34.50	34.32	34.36
VirF	Transcritional regulator	28.40	27.98	28.05	28.09	27.64	27.85	28.15	27.87	30.72	30.83	30.53	30.20	30.38	30.82	30.82	30.59	30.78	30.38	30.75
YscB	Chaperone	32.16	32.13	31.80	31.83	31.32	31.94	32.04	31.72	33.24	33.89	34.02	33.15	33.39	33.81	33.78	33.44	33.60	33.57	33.69
SctP	Needle length measurement	31.97	31.82	31.87	31.77	31.36	31.48	31.89	31.55	32.45	32.89	32.98	32.61	32.60	33.09	32.56	32.78	32.68	32.74	32.83
SctU	Export apparatus	25.54	25.70	25.59	24.42	25.12	25.62	25.61	25.14	29.83	30.17	30.30	30.06	30.01	30.40	29.84	30.15	30.01	30.13	30.15
SycO	Chaperone	30.57	30.29	30.55	29.98	29.96	29.90	30.48	29.95	30.75	30.74	31.05	29.89	30.30	31.12	31.09	30.29	30.74	30.49	30.88
YlpA	Lipoprotein	28.70	28.47	28.70	28.28	27.96	27.93	28.63	28.07	31.19	31.62	31.43	31.65	31.54	31.28	31.23	31.36	31.42	31.54	31.29
YadA	Adhesin	29.92	29.36	30.17	29.27	29.14	29.37	29.86	29.27	35.83	35.59	35.76	35.58	35.64	35.86	35.63	35.56	35.72	35.66	35.69
SctI	Needle adaptor protein	30.72	30.46	30.69	30.23	29.62	29.45	30.63	29.81	33.12	33.43	33.50	33.31	33.44	33.52	33.22	33.32	33.29	33.42	33.36
SctD	Inner membrane ring protein	29.61	29.18	29.57	28.50	27.80	28.33	29.47	28.24	32.61	32.84	32.85	32.58	32.74	32.81	32.83	32.73	32.73	32.73	32.79
LcrG	Chaperone	31.57	31.56	31.60	25.14	30.02	30.82	31.57	29.91	33.74	33.96	33.63	33.91	33.74	33.94	33.83	34.16	33.85	33.76	33.98

Suppl. Table 2: Number of measured trajectories and replications in single particle tracking experiments in this study

Strain	Number of experiments (*n*)	Analyzed trajectories	Used in figure
pYV$^-$ PAmCherry	3	19377	Suppl. 4
pYV$^-$ PAmCherry-SctQ	3	26571	1
pYV$^-$ PAmCherry-SctQ + YopO/SycO	4	60426	1
pYV$^-$ PAmCherry-SctL	3	36040	1
pYV$^-$ PAmCherry-SctL + YopO/SycO	3	45426	1
pYV$^-$ PAmCherry-SctN	3	22428	1
pYV$^-$ PAmCherry-SctN + YopO/SycO	3	18747	1
PAmCherry-SctQ Δeffectors	3	18905	2
PAmCherry-SctQ	5	43071	2
PAmCherry-SctQ secreting	3	42104	2
PAmCherry	4	12109	Suppl. 10
PAmCherry secreting	4	15567	Suppl. 10
PAmCherry-SctQ Δ*sctD* Δeffectors	7	79876	3
PAmCherry-SctQ Δ*sctD*	5	21861	3
PAmCherry-SctQ Δ*sctK* Δeffectors	4	29444	3
PAmCherry-SctQ Δ*sctK*	4	34747	3
PAmCherry-SctQ Δ*sctL* Δeffectors	4	35716	3
PAmCherry-SctQ Δ*sctL*	7	55558	3

Suppl. Table 3: Label-free mass spectrometry quantification of cellular amounts of the indicated T3SS substrates and machinery components.

To determine the ratio of SctQ and its potential binding partners, we quantified the cellular concentrations of T3SS export substrates (encompassing both translocator proteins and effector proteins) and the sorting platform components by label-free mass spectrometry. The table lists the intensity values for all indicated T3SS components in the listed strains and conditions. # pept., number of total peptides detected. Three biological replicates, as indicated. See Material and Methods for details. As expected, the concentration of effectors in the Δeffectors strain was very low (only YopQ, an effector with no known virulence function that is thought to act as a regulator of translocation rate [6] is still present in the Δeffectors strain) and the amount of effectors drastically increased under secreting conditions in the wild-type strain, in comparison to non-secreting conditions. The amount of translocators was very similar in both strains and further increased under secreting conditions. Whether the fraction of the Δeffectors tracks with an mjd in the "single pods with effectors" range (Fig. 2d) is caused by the binding of these translocators to the pod structure, is unclear and difficult to test in native settings, as deletions of the translocators causes a deregulation of the system. Expression of SctQ and the other sorting platform components was less strongly upregulated in secreting bacteria, in agreement with previous results [7].

			**Δeffectors (dHOPEMTasd)**			**wild-type non-secreting**			**wild-type secreting**			**Δeffectors**	**WT non-secreting**	**WT secreting**
Protein ID	Protein description	# pept.	Replic. 1	Replic. 2	Replic. 3	Replic. 1	Replic. 2	Replic. 3	Replic. 1	Replic. 2	Replic. 3	Mean value	Mean value	Mean value
	**Effectors**													
AAD16803.1	Yop effector protein kinase YopO	52	4.72E+07	4.35E+07	4.81E+07	5.80E+07	4.57E+07	4.41E+07	1.66E+09	2.35E+09	1.27E+09	4.63E+07	4.93E+07	1.76E+09
AAD16805.1	Yop effector YopP	20	4.57E+06	3.05E+07	3.27E+04	1.38E+05	9.21E+04	3.21E+04	1.40E+09	1.98E+09	7.50E+08	1.17E+07	8.76E+04	1.38E+09
AAD16807.1	Yop effector YopQ	17	5.40E+08	4.81E+08	3.70E+08	1.50E+08	1.78E+08	1.69E+08	2.47E+09	2.92E+09	1.78E+09	4.64E+08	1.66E+08	2.39E+09
AAD16808.1	Yop effector YopT	6	3.43E+06	5.73E+06	8.51E+06	5.26E+06	5.76E+06	3.79E+06	2.26E+08	2.13E+08	1.41E+08	5.89E+06	4.94E+06	1.93E+08
AAD16811.1	Yop effector YopM	3	8.00E+05	6.55E+05	9.45E+06	1.61E+08	2.05E+08	6.37E+08	6.00E+09	7.13E+09	4.90E+09	3.64E+06	3.34E+08	6.01E+09
AAD16847.1	Yop effector YopH	45	8.60E+04	5.54E+05	2.52E+05	2.58E+08	2.35E+08	1.48E+08	1.22E+10	1.48E+10	8.83E+09	2.98E+05	2.13E+08	1.20E+10
AAD16850.1	Yop effector YopE	15	9.62E+06	1.08E+07	9.04E+06	1.61E+09	1.61E+09	1.57E+09	1.43E+10	1.91E+10	1.11E+10	9.82E+06	1.60E+09	1.49E+10
	**Translocators**													
AAD16812.1	Translocator SctB/YopD	37	1.76E+09	1.66E+09	1.21E+09	8.06E+08	7.92E+08	8.43E+08	1.77E+10	2.25E+10	1.38E+10	1.54E+09	8.14E+08	1.80E+10
AAD16813.1	Translocator SctD/YopB	25	6.44E+08	6.99E+08	5.62E+08	2.91E+08	2.92E+08	3.08E+08	8.70E+09	1.08E+10	7.06E+09	6.35E+08	2.97E+08	8.86E+09
AAD16815.1	Translocator SctA/LcrV	30	8.88E+08	7.84E+08	5.75E+08	4.02E+08	3.94E+08	4.31E+08	5.11E+09	4.81E+09	4.41E+09	7.49E+08	4.09E+08	4.78E+09
	**Other exported proteins**													
AAD16820.1	Regulator / secreted protein YscX	9	2.74E+08	2.64E+08	1.58E+08	1.08E+08	1.24E+08	1.06E+08	6.55E+08	7.12E+08	5.00E+08	2.32E+08	1.13E+08	6.23E+08
AAD16823.1	Gatekeeper SctW/YopN	20	9.99E+08	8.18E+08	8.11E+08	5.15E+08	5.46E+08	5.65E+08	2.28E+09	2.53E+09	1.50E+09	8.76E+08	5.42E+08	2.11E+09
AAD16825.1	ATPase stalk protein SctO/YscO	5	4.54E+07	4.41E+07	5.19E+07	1.92E+07	2.53E+07	2.81E+07	1.15E+08	7.83E+07	1.03E+08	4.71E+07	2.42E+07	9.87E+07
AAD16826.1	Ruler / needle length regulator SctP/YscP	29	4.54E+08	4.48E+08	3.20E+08	2.64E+08	2.59E+08	2.66E+08	1.10E+09	1.20E+09	8.65E+08	4.07E+08	2.63E+08	1.06E+09
AAD16839.1	Needle subunit SctF/YscF	10	1.49E+09	1.27E+09	9.11E+08	7.34E+08	7.41E+08	6.80E+08	3.68E+09	3.72E+09	3.48E+09	1.23E+09	7.18E+08	3.62E+09
AAD16841.1	Secreted protein YscH/YopR	7	2.54E+08	1.85E+08	1.63E+08	1.26E+08	1.21E+08	1.32E+08	6.88E+08	6.93E+08	4.55E+08	2.01E+08	1.26E+08	6.12E+08
AAD16846.1	Regulatory protein YscM1	9	1.20E+09	1.25E+09	8.63E+08	4.75E+08	4.75E+08	5.03E+08	7.58E+07	6.75E+07	4.51E+07	1.10E+09	4.84E+08	6.28E+07
AAD16867.1	Regulatory protein YscM2	7	6.44E+08	6.66E+08	4.37E+08	5.56E+08	6.00E+08	6.88E+08	1.36E+08	1.59E+08	8.93E+07	5.82E+08	6.15E+08	1.28E+08
	**Sorting platform proteins and ATPase**													
AAD16827.1	C-ring / pod subunit SctQ/YscQ	13	6.31E+08	7.22E+08	1.04E+09	4.48E+08	4.22E+08	3.68E+08	1.36E+09	1.43E+09	1.86E+09	7.99E+08	4.13E+08	1.55E+09
AAD16844.1	Accessory / adapter protein SctK/YscK	11	2.21E+08	1.67E+08	2.38E+08	1.13E+08	1.32E+08	9.97E+07	4.99E+08	4.92E+08	5.37E+08	2.09E+08	1.15E+08	5.09E+08
AAD16845.1	Stator / neg. ATPase regulator SctL/YscL	13	4.89E+08	4.08E+08	3.52E+08	2.00E+08	2.24E+08	2.20E+08	1.01E+09	9.53E+08	8.56E+08	4.16E+08	2.15E+08	9.41E+08
AAD16824.1	ATPase SctN/YscN	28	1.99E+08	2.35E+08	3.68E+08	1.33E+08	1.28E+08	1.11E+08	6.42E+08	6.82E+08	8.08E+08	4.65E+08	5.68E+08	4.88E+08

Total												
**Effectors total**		**6.06E+08**	**5.73E+08**	**4.46E+08**	**2.24E+09**	**2.28E+09**	**2.57E+09**	**3.83E+10**	**4.85E+10**	**2.88E+10**	**5.42E+08**	**2.36E+09**	**3.85E+10**
**Translocators total**		**3.29E+09**	**3.15E+09**	**2.34E+09**	**1.50E+09**	**1.48E+09**	**1.58E+09**	**3.15E+10**	**3.81E+10**	**2.53E+10**	**2.93E+09**	**1.52E+09**	**3.16E+10**
**Other exported proteins**		**5.36E+09**	**4.95E+09**	**3.71E+09**	**2.80E+09**	**2.89E+09**	**2.97E+09**	**8.73E+09**	**9.16E+09**	**7.04E+09**	**4.67E+09**	**2.88E+09**	**8.31E+09**

normalized relative to SctQ level

**Effectors total**	**0.68**	**5.73**	**24.89**
**Translocators total**	**3.67**	**3.68**	**20.44**
st. dev. (effectors)	*0.11*	*0.44*	*6.38*
st. dev. (translocators)	*0.64*	*0.13*	*4.14*

Suppl. Table 4: List of plasmids and strains used in this study

**Plasmid**	**Genotype**	**Reference**
pACYC184	p15A-derived expression vector	Invitrogen
pBAD-His B	pBR322-derived expression vector	Invitrogen
pCDFduet	Expression vector for coexpression of two genes	Novagen
pET24b	Expression vector	Novagen
pKNG101	*oriR6K sacBR$^+$ oriTRK2 strAB$^+$* (suicide vector for allelic exchange)	[8]
pAD164	*pKNG101-ΔsctD*	[4]
pAD443	*pKNG101-pamch1-sctQ*	[9]
pAD484	*pKNG101-ΔsctK*	this study
pAD765	*pACYC184::pamch1-sctL*	this study
pAD766	*pACYC184::pamch1-sctN*	this study
pAD833	*pET24b::sctQ-flag*	this study
pAD837	*pET24b::egfp-flag*	this study
pAD839	*pCDFduet::his-yopO,sycO*	this study
pAD844	*pET24b::sctQ-his*	this study
pAD848	*pET24b::egfp-his*	this study
pCB041	*pKNG101-miniturbo-flag-sctQ*	this study
pCB055	*pBAD::egfp-miniturbo-flag*	this study
pKP012	*pKNG101-sycH-egfp-flag*	this study
pKP024	*pKNG101-yopH-egfp-flag*	this study
pSI51	*pKNG101-ΔsctL*	[4]
pSW039	*pBAD::pamch1*	this study
pSW040	*pBAD::sycO,yopO-flag*	this study
pSW042	*pACYC184::pamch1-sctQ*	this study

**Strain**	**Genotype**	**Reference**
MRS40	Wild-type *Y. enterocolitica* E40 Δ*blaA*	[10]
IML421*asd* (ΔHOPEMTasd) (Δeffectors)	MRS40 *yopO*$_{Δ12\text{-}427}$ *yopE*$_{21}$ *yopH*Δ$_{11\text{-}352}$ *yopM*$_{23}$ *yopP*$_{23}$ *yopT*$_{135}$ Δ*asd*	[11]
pYV$^-$	MRS40 cured of virulence plasmid	laboratory collection
AD4443	IML421*asd pamch1-sctQ*	[9]
AD4460	IML421*asd pamch1-sctQ* Δ*sctD*	this study
AD4509	IML421*asd pamch1-sctQ* Δ*sctL*	this study
AD4553	MRS40 *pamch1-sctQ*	this study
CB4006	MRS40 *miniturbo-flag-sctQ*	this study
KP001	MRS40 *yopH-egfp-flag*	this study
KP018	MRS40 *sycH-egfp-f*	this study
SW4019	MRS40 *pamch1-sctQ* Δ*sctL*	this study
SW4020	MRS40 *pamch1-sctQ* Δ*sctD*	this study
SW4029	MRS40 *pamch1-sctQ* Δ*sctK*	this study
SW4030	IML421*asd pamch1-sctQ* Δ*sctK*	this study

Suppl. Table 5: List of oligonucleotides used in this study

Name	Oligonucleotide sequence	Used for plasmid(s)
AD931	GACTTTCGAATTAGAATTCACCAGATCTTCCCTTATCATCGTCGTCCTTGTAGTCACCCCATTCCCGCTCCAACCG	pSW040
AD984	GACTGGTCTCCCATGGTGAGCAAGGGCGAGGAG	pSW039
AD985	GACTGAATTCTTACTTGTACAGCTCGTCCATGC	pSW039
AD1039	GACTGGTCTCCCATGATTAACACCACCTTTACTGAGCTA	pSW040
AD1040	GACTGGATCCTTGACTGAATGAGTTTGAGATCTGGTGCAG	pSW042
AD1047	GACTGTCGACTCATGAAATCGTAACCTCTGTCA	pSW042
AD1321	TATAGGTCTCGGGCCCACTCGCTTCACTGGTAGATCA	pKP024
AD1322	GTCCTTGTAGTCACCTCCCAATTGTATAAGATCTACCACCAGAGCCGCCCGAACCTCCGCTATTTAATAATGGTCGCCCTTGT	pKP024
AD1323	CAATTGGGAGGTGACTACAAGGACGACGATGATAAGTAAATTACTAAGAGATATACACCACTTTGCC	pKP024
AD1324	AAGGTCTCTCTAGAGCTGTTACTCTTTCTTGTGAACCC	pKP024
AD1330	GACTGGATCCTTGACTGAATGTCACAAACTTGTCAAACAGGT	pAD765
AD1331	GACTGTCGACTTATTCCTCTTCTGTAACTTTCATTTGTCC	pAD765
AD1332	GACTTCATGATTGACTGAATGCTCTCACTAGATCAGATACCTCA	pAD766
AD1333	GACTGTCGACTCATTGGGTCAGCGTCTCCA	pAD766
AD1528	GACTGGGCCCAGCATCTGGGAAGGAGAGAAAT	pKP012
AD1529	ACTGAGATCTGCCACCAGACCCGCCCGAACCACCAACCAGTAAATGAGATGATGAAGG	pKP012
AD1530	GGGTCTGGTGGCAGATCTCAGTCAATTGTAATTCTATAAAAGAAAAACGTAC	pKP012
AD1531	GACTTTCTAGAGAAAGCAAAGGAAGTTCAGCGG	pKP012
AD1789	TATAGGTCTCCCATGTCTAGCCATCACCATCACCATCATCACCATGGAGGGTCAGGAGGTAAAATCATGGGAACTATGCCACC	pAD839
AD1790	TATAGAATTCACATCCATTCCCGCTCCAACC	pAD839
AD1797	TATACCATGGCTAGCAGTTTGTTAACCTTGCCACAAGC	pAD833, pAD844
AD1798	TATAGAATTCACTTATCATCGTCGTCCTTGTAGTCACCTCCTGACCCTCCTGAAATCGTAACCTCTGTCAGGC	pAD833
AD1799	TATAGAATTCAATGGTGATGATGGTGATGGTGATGACCTCCTGACCCTCCTGAAATCGTAACCTCTGTCAGGC	pAD844
AD1806	TATACCATGGCTAGCGTGAGCAAGGGCGAGGAG	pAD837, pAD848
AD1807	TATAGAATTCACTTATCATCGTCGTCCTTGTAGTCACCTCCTGACCCTCCCTTGTACAGCTCGTCCATGC	pAD837
AD1808	TATAGAATTCAATGGTGATGATGGTGATGGTGATGACCTCCTGACCCTCCCTTGTACAGCTCGTCCATGC	pAD848
AD1809	TATACATATGATTAACACCACCTTTACTGAGCTACT	pAD839
AD1810	TATACTCGAGTCATCCCCATTTAACCGATTGAGTA	pAD839

Suppl. Table 6: Assignment of raw data of proteomics files

Assignment of raw mass spectrometry proteomics data files deposited to the ProteomeXchange Consortium via the PRIDE partner repository [12] with the dataset identifier PXD044214.

**Figures**	**Method**	**Name of raw files for indicated strains** (unique numbers in bold font)
Table 1, Suppl. Table 1	Eluate of streptavidin purification after proximity labeling	miniTurbo-SctQ: "YscQPL-6-**4-6"**<br>miniTurbo-EGFP: "YscQPL-6-**7-9**"
Table 1, Suppl. Table 1	Total cell lysate for comparison	miniTurbo-SctQ: "YscQPL-6-**13-15"**<br>miniTurbo-EGFP: "YscQPL-6-**16-18**"<br>untagged wild-type: "YscQPL-6-**10,12**"
Suppl. Fig. 3	Eluate of co-immunoprecipitation of PAmCherry-SctQ	PAmCh-SctQ: "PAmCh6-**7-9**re"<br>PAmCh control: "PAmCh6-**1-3**re"
Suppl. Fig. 3	Eluate of co-immunoprecipitation of PAmCherry-SctL	PAmCh-SctL: "PAmCh3-**2,5,8"**<br>PAmCh control: "PAmCh3-**1**re,**4,7"**
Suppl. Fig. 3	Eluate of co-immunoprecipitation of PAmCherry-SctN	PAmCh-SctN: "PAmCh6-**16-18**re"<br>PAmCh control: "PAmCh6-**10-12**re"
Suppl. Table 3	Total cell lysate	Δeffectors: "SW-TC-2-**10-12**"<br>wild-type non-secreting: "SW-TC-2-**4-6**"<br>wild-type non-secreting: "SW-TC-2-**1-3**"

Suppl. Videos 1-3: Examplary raw microscopy data of single particle tracking photoactivated localization microscopy:

Suppl. Video 1:

Examplary raw microscopy data of single particle tracking photoactivated localization microscopy for PAmCherry-SctQ in strain delta-effectors, non-secreting conditions (d_eff_nscr.tif). Frame rate 15 ms, file size 3.1 GB.

Suppl. Video 2:

Examplary raw microscopy data of single particle tracking photoactivated localization microscopy for PAmCherry-SctQ in wild-type strain, non-secreting conditions (WT_nscr.tif). Frame rate 15 ms, file size 2.9 GB.

Suppl. Video 3:

Examplary raw microscopy data of single particle tracking photoactivated localization microscopy for PAmCherry-SctQ in wild-type strain, secreting conditions (WT_scr.tif). Frame rate 15 ms, file size 2.8 GB.

Supplementary Videos 1-3 can be downloaded at the Max Plack Society data repository under the URL

https://edmond.mpdl.mpg.de/privateurl.xhtml?token=0b3ec8a9-ac00-44bb-bc7b-33694b4f4eb7

and are accessible under the doi:10.17617/3.HMABQ2 upon publication of the manuscript. All videos are in the original 14-bit format and can be viewed with software such as FIJI/ImageJ.

Suppl. Video 4: Examplary part of single particle tracking photoactivated localization microscopy for PAmCherry-SctQ in wild-type strain, non-secreting conditions with adjusted contrast in 8-bit format. Frame rate 15 ms, file size 17 MB.

Suppl. Text 1: Predicting diffusion behavior of the T3SS sub-complexes

It can be difficult to predict diffusion directly, e.g., by calculating a diffusion coefficient or an expected jump distance per frame for a molecule diffusing in the cytosol of a bacterial cell. First, molecules differ in their diffusion from pure Brownian diffusion because of their shape, surface charges, and interaction sites. In addition, their diffusion is confined to the small bacterial volume and can vary spatially in the cytosol due to its heterogeneous molecular composition, which leads to different densities, viscosities, and charges within the cytosol. These molecular crowding factors define the local environment of each molecule and can individually impede its mobility, broadening the observed distributions. Finally, the observed diffusion in sptPALM imaging can be affected by the temporal resolution of the technique, which is unable to resolve binding and interaction times at sub-frame rates. This is reflected in apparently slower diffusion coefficients than expected for free diffusion as the molecular binding state cannot be directly resolved. For all these reasons, the experimental values for the diffusion coefficient and jump distance often differ significantly from the theoretical values based on molecular weights and the Einstein-Stokes relationship [13].

In contrast, based on the assumption that the various influences on diffusive behavior will be to a large extent similar in between the T3SS sub-complexes due to their high structural and molecular similarity, we can predict their expected diffusion behavior in a relative manner based on their mass ratios as given by $mjd_2 = \left(\frac{m_1}{m_2}\right)^{1/6} * mjd_1$ [based on Einstein-Stokes D = $k_BT/(6\pi\eta R_H)$, the hydrodynamic radius $R_H = \sqrt[3]{\frac{M/N_A}{4\,\pi\rho}}$ and the relation between jump distances and diffusion coefficient as given by $mjd^2 = \sigma^2\pi/2$ and $\sigma^2 = 2D\tau$ for 2D Brownian free diffusion], e.g. using the mass ratio between a single pod with and without effectors or a full cytosolic sorting platform consisting of six single pods. As the reference value mjd_1, we used the mjd value of 272.5 nm of the main peak of SctQ-PAmCherry diffusion in the effector-less strain (Fig. 2d) and assumed that it represents the diffusion of individual pods with the stoichiometry $SctK(Q(Q_C)_2)_4L_2N$. Using this, we thus could calculate the expected mean jump distances of the other sub-complexes based on the molecular weights of T3SS components, effectors and chaperones. We determined the molecular weights based on the protein sequences of the *Y. enterocolitica* virulence plasmid pYVe227 (GenBank accession AF102990.1) and the sequences of the respective labeled proteins. The molecular weight of an average effector-chaperone complex (65.8 kDa) was based on the average of the molecular weights of effectors YopH, O, P, E, M, T in complexes with dimers of their respective chaperones. The molecular weight of complexes were calculated by adding the molecular weight of the indicated copy number of the respective proteins; see [14] for additional possible stoichiometries. All expected mjd values can be found in Table 1.

Notably, the increasing mobility observed for SctL<SctQ<SctN in the strain lacking other injectisome components (Fig. 1bc) matches the expected order, based on the available data for the multimerization state of the tested proteins (107.2 kDa for SctL (PAmCherry-$SctL_2$)[5,14–17], 82.5 kDa for SctQ (PAmCherry-SctQ-$SctQ_{C,2}$ complex)[18,19], and 77.3 kDa for SctN (PAmCherry-SctN).

Suppl. Text 2: Variety of T3SS sub-complexes

It was shown earlier [5,14,20] that the cytosolic T3SS components SctK, Q, L, N exist in a broad array of subcomplex compositions, which result in different protein mobility, even without taking into account the possibility of substoichiometric binding of effectors to the respective components. While we focus on the most prominent mobility peaks in our analysis, we therefore expect a variety of subcomplexes and resulting broad mobility distribution. The presence of cytosolic sorting platform proteins is not due to rapid protein turnover and replacement (Suppl. Fig. 5).

Suppl. Text 3: Detection rate of PAmCherry-SctQ molecules and quantification of injectisomes per bacterium

A key advantage of sptPALM in live bacteria is the possibility to directly observe the impact of the activation of secretion on the sorting platform and its interactions. This allows to perform *in vivo* biochemical measurements in different conditions relevant for bacterial cell physiology and virulence. While we used a stable genetic fusion of PAmCherry to SctQ (Suppl. Fig. 1), allowing to assume a complete labeling of SctQ protein, the detection of fluorescent emitters is limited by different factors, such as the percentage of incompletely matured chromophores or non-photoactivated or prematurely photobleached PAmCherry molecules [21]. In addition, by far not all PAmCherry molecules are read out during sptPALM image acquisition, which is performed for a rather short time to avoid phototoxic effects (see materials and methods for details). For these reasons, quantitative analysis of foci has to be corrected for under-counting of PAmCherry-labeled SctQ molecules. Quantification of single-molecule trajectories per injectisome resulted in similar numbers of SctQ molecules for both non-secreting and secreting conditions, with on average 2.0 ± 1.3 (median = 2) and 1.8 ± 1.0 (median = 2) SctQ trajectories, respectively (Suppl. Fig. 7a). As expected, these values are highly below the expected value of 24 SctQ molecules per injectisome [5,9,16]. To determine the probability of not detecting any SctQ molecule per injectisome, we performed a binomial distribution fit analysis (materials and methods, Suppl. Fig. 7b) and found a detection probability pk of 0.060 ± 0.001, with k being the number of successes (Suppl. Fig. 7b). This allowed us to determine that the probability of not detecting any of the 24 PAmCherry-SctQ molecules per injectisome is 0.229 ± 0.006, resulting in a correction factor of 1 – 0.229 = 0.771. Correction of our injectisome-per-cell quantification resulted in 5.1 ± 1.7 injectisomes under non-secreting, and 17.9 ± 5.4 injectisomes under secreting conditions, respectively (Fig. 2bc). The higher number of injectisomes under secreting conditions is in line with an increased expression and assembly of injectisome components upon activation of type III secretion by low extracellular calcium levels[7,22]. While the upregulation upon secretion is slightly higher in our study, these results are in line with an earlier study combining fluorescence microscopy and cryo-electron tomography in *Y. enterocolitica*[7] and the finding that the copy number of the virulence plasmid in *Y. pseudotuberculosis* increased by a factor of two under secreting conditions[23].

Suppl. Text 4: Jump distances as a measure of particle movement

For our work, we use jump distances (JDs) as a measure of protein movement, because this does not require any assumptions, e.g. on the type of motion. It is simply a measure on how far the particle moved in two consecutive camera frames. Furthermore, as we only look at one-step distances, we lower the influence of the cellular confinement on this measurement, a typical source of error for movement analyses in small bacterial volumes.

Simply plotting individual JDs, however, ignores critical information that was gained during the tracking step, namely that JDs from the same trajectory segment belong to the same subpopulation. We incorporate this information by instead plotting the mean jump distance (MJD) for each trajectory segment weighted by the number of jumps. In the present study, this was crucial to clearly separate the subpopulations. The downside of this approach, however, is that, even if the individual JDs in a subpopulation follow a known distribution, the (weighted) MJDs do not. Fitting a (mixture) distribution to the MJD plot to characterize the subpopulations is therefore not possible.

Importantly though, the weighted mean of the MJDs of a subpopulation is identical to the mean of its JD distribution. Furthermore, for a sufficiently large number of jumps per trajectory segment, the peak of a weighted MJD distribution converges to its mean. Thus, for JD or weighted MJD plots encompassing several subpopulations, each MJD peak of a subpopulation can be a used to estimate the mean of its original JD distribution. This is especially important in cases (like in this manuscript) where subpopulations overlap considerably, even for typical sptPALM data of rather limited trajectory length, where the MJD peak is usually slightly below the mean.

<u>Estimating the diffusion coefficient D from MJD plots</u>

Assuming the particles in a subpopulation are in fact diffusing Brownian, their JDs follow a Rayleigh distribution with a mean at $\sigma\sqrt{(\pi/2)}$, where σ is the standard deviation characterizing the *apparent* diffusion (see below). By using MJD_{peak}, the peak of its weighted MJD distribution, as an approximation for the mean of the JD distribution of the subpopulation, we can estimate σ as

$$\sigma \approx MJD_{peak}/\sqrt{(\pi/2)}$$

The diffusion coefficient D, in turn, can be estimated from σ by the following well-known relation typically used to estimate diffusion in single-particle tracking:

$$\sigma^2 = 2Dt + 2\sigma_{mes}^2 - 2/3\ Dt$$

with σ_{mes} being the static error as given by the localization precision and -2/3Dt being the dynamic error (4RDt) that arises from the particle motion during the exposure time of the camera and which can be calculated by averaging all the positions the particle takes while the camera shutter is open, in our case for a full frame time exposure yielding R = 1/6 [24–26].

Putting this together, we can estimate the diffusion coefficients D (µm²/s) as given in Table 2 using the following relation

$$D \approx 3/2(MJD_{peak}^2\pi^{-1} - \sigma_{mes}^2)\tau^{-1}$$

using the frame rate τ = 15 ms used in the measurements for this study and the localization precision σ_{mes} with a median of 15.6 nm, average of 16.5 +/- 4.4 nm, obtained for this study. Taking the immobile MJD peak of injectisome-bound complexes of 40.5 nm (Suppl. Figure 11) as an example, this yields an almost ideally zero $D_{immobile}$ of 0.02 µm²/s as both, static and dynamic errors are corrected for.

Suppl. Text 5: Composition and stoichiometry of T3SS sorting platform pods

The presence of soluble cytosolic sorting platform subcomplexes has been shown in different bacteria, including *Shigella flexneri* [27], *Salmonella enterica* [16,28] and *Yersinia enterocolitica* [5,9,20,29], and by various methods including biochemical studies, fractionation, fluorescence microscopy, and single particle tracking. *In situ* structures of injectisomes [15,30–32], SAXS structure analysis and native mass spectrometry [14] and fluorescence microscopy-based stoichiometry analyses [5,14,16] indicate that the sorting platform proteins predominantly form "pod" structures, which most likely contain one SctK, 2-4 SctQ, 4-8 $SctQ_C$ [a], 2 SctL, and one SctN.

Our observation of a 2:1 stoichiometry of injectisome-bound SctQ:SctL (Extended Data Fig. 3) supports an $SctK\text{-}Q(Q_C)_2)_4\text{-}L_2\text{-}N$ complex. The mobility measurements in this study suggest that effectors are bound to such pod structures, as well as $SctQ(Q_C)_2$ and $SctL_2$ subcomplexes in the bacterial cytosol (Table 2).

The activation of secretion also led to a pronounced upregulation of effector production (Fig. 2a, Suppl. Table 3) [33–35]. Accordingly, the fraction of slower complexes, which we interpret as effector-bound complexes, was further increased under secreting conditions in our experiments (Fig. 2d). The molecular weight corresponds to a - most likely temporary - complete sorting platform complex consisting of six pod structures (Table 2). While this matches the hexameric nature of the ATPase SctN (an increased hexamerization tendency of which might cause the emergence of these large complexes under secreting conditions) [36–38] and the hexameric structure of certain effector/chaperone complexes [39], the role of these complexes is currently unclear. The absence of one-step fluorescence recoveries in photobleaching experiments [9] suggests that these large assemblies do not directly bind to the injectisome. These results are in agreement with earlier findings for the *Salmonella* SPI-1 T3SS, where SctQ formed very large complexes, even in the absence of the T3SS membrane ring components [28]. It is also consistent with the observation that SPI-1 effector proteins were localized throughout the cytosol, but also formed larger clusters [16].

A question that remains open is the role and time point of action of the ATPase SctN. Similar to our finding of exchange of sorting platform proteins at the injectisome, exchange of complexes containing one copy of the flagellar ATPase FliI (a homolog of SctN) has been observed in the bacterial flagellum [40]. While SctN has been shown to interact with the sorting platform in various studies [5,14,27], its absence has a comparably low impact on both soluble and injectisome-bound sorting platform complexes [5,15]. Further studies are needed to determine if the proposed role of the ATPase in removing chaperones from the effectors [41] occurs before, at the same time, or after the binding of the effectors to the sorting platform. The finding that T3SS chaperones can bind to the sorting platform in the absence of their effectors [28,42] argues for one of the latter options.

---

[a] $SctQ_C$ was not detected in fully assembled sorting platforms in the *Salmonella* Typhimurium SPI-1 T3SS [43], which was attributed to an overlap of $SctQ_C$ and SctL binding sites in the full-length SctQ. However, $SctQ_C$ was found to be a part of sorting platform substructures (including SctL) in soluble complexes of the SPI-1 sorting platform [14] and of injectisome-bound complete sorting platform in *Yersinia enterocolitica* [9].

Suppl. Text 6: Calculation of SctQ exchange rate at single injectisomes

The exchange rate $r_{ex(SctQ)}$ in SctQ proteins per injectisome is a function of the number of SctQ proteins per injectisome ($\#_{SctQ}$) and the half-time of recovery of SctQ in photobleaching experiments ($t_{½}$), corresponding to the exchange of half of the bleached SctQ proteins by proteins from the unbleached cytosolic pool.

Using the values $\#_{SctQ}$ = 24 [5,9,16] and $t_{½}$ = 68.2 s [9], we can calculate

$$r_{ex(SctQ)} = \frac{\#_{SctQ}}{t_{1/2}\ln(2)} = \frac{24}{68.2s\ln(2)} \approx 0.51\ s^{-1}$$

Suppl. Data 1: Raw data and statistical analysis of sptPALM measurements in *Y. enterocolitica*

Fractions of measured trajectories in indicated mjd ranges (center of bin given in nm) and statistical analysis. Three color gradient coding applied to visualize single values (green = 0, via yellow to, red = highest value) and cumulative values (blue = 0, via white = 0.5 to red = 1). Tab a) relates to Fig. 1, b) Fig. 2, c) Fig. 3, d) Suppl. Fig. 10.

Suppl. Data 2: Source Data file for Supplementary Figures

Source data for Supplementary Figures

References for Supplementary Information

1. Bertani, D., Oppenheim, A. B. & Narberhaus, F. An internal region of the RpoH heat shock transcription factor is critical for rapid degradation by the FtsH protease. *FEBS Lett.* **493**, 17–20 (2001).
2. Nagashima, K. *et al.* Degradation of Escherichia coli RecN aggregates by ClpXP protease and its implications for DNA damage tolerance. *J. Biol. Chem.* **281**, 30941–30946 (2006).
3. Gupta, M. *et al.* Global Protein-Turnover Quantification in Escherichia coli Reveals Cytoplasmic Recycling under Nitrogen Limitation. *bioRxiv* 2022.08.01.502339 (2023) doi:10.1101/2022.08.01.502339.
4. Diepold, A. *et al.* Deciphering the assembly of the Yersinia type III secretion injectisome. *EMBO J.* **29**, 1928–40 (2010).
5. Diepold, A. *et al.* A dynamic and adaptive network of cytosolic interactions governs protein export by the T3SS injectisome. *Nat. Commun.* **8**, 15940 (2017).
6. Dewoody, R. S., Merritt, P. M. & Marketon, M. M. Regulation of the Yersinia type III secretion system: traffic control. *Front. Cell. Infect. Microbiol.* **3**, 4 (2013).
7. Kudryashev, M. *et al.* Yersinia enterocolitica type III secretion injectisomes form regularly spaced clusters, which incorporate new machines upon activation. *Mol. Microbiol.* **95**, 875–884 (2015).
8. Kaniga, K., Delor, I. & Cornelis, G. R. A wide-host-range suicide vector for improving reverse genetics in Gram-negative bacteria: inactivation of the blaA gene of Yersinia enterocolitica. *Gene* **109**, 137–41 (1991).
9. Diepold, A., Kudryashev, M., Delalez, N. J., Berry, R. M. & Armitage, J. P. Composition, Formation, and Regulation of the Cytosolic C-ring, a Dynamic Component of the Type III Secretion Injectisome. *PLOS Biol.* **13**, e1002039 (2015).
10. Sory, M.-P., Boland, A., Lambermont, I. & Cornelis, G. R. Identification of the YopE and YopH domains required for secretion and internalization into the cytosol of macrophages, using the cyaA gene fusion approach. *Proc. Natl. Acad. Sci. U. S. A.* **92**, 11998–12002 (1995).
11. Kudryashev, M. *et al.* In situ structural analysis of the Yersinia enterocolitica injectisome. *Elife* **2**, e00792 (2013).
12. Perez-Riverol, Y. *et al.* The PRIDE database resources in 2022: a hub for mass spectrometry-based proteomics evidences. *Nucleic Acids Res.* **50**, D543–D552 (2022).
13. Bellotto, N. *et al.* Dependence of diffusion in Escherichia coli cytoplasm on protein size, environmental conditions and cell growth. *Elife* **11**, 2022.02.17.480843 (2022).
14. Bernal, I. *et al.* Molecular Organization of Soluble Type III Secretion System Sorting Platform Complexes. *J. Mol. Biol.* **431**, 3787–3803 (2019).
15. Hu, B., Lara-Tejero, M., Kong, Q., Galán, J. E. & Liu, J. In Situ Molecular Architecture of the Salmonella Type III Secretion Machine. *Cell* **168**, 1065-1074.e10 (2017).
16. Zhang, Y., Lara-Tejero, M., Bewersdorf, J. & Galán, J. E. Visualization and characterization of individual type III protein secretion machines in live bacteria. *Proc. Natl. Acad. Sci. U. S. A.* **114**, 6098–6103 (2017).
17. Soto, J. E. & Lara-Tejero, M. The sorting platform in the type III secretion pathway: From assembly to function. *BioEssays* 1–11 (2023) doi:10.1002/bies.202300078.
18. Bzymek, K. P., Hamaoka, B. Y. & Ghosh, P. Two translation products of Yersinia yscQ assemble to form a complex essential to type III secretion. *Biochemistry* **51**, 1669–77 (2012).
19. McDowell, M. A. *et al.* Characterisation of Shigella Spa33 and Thermotoga FliM/N reveals a new

model for C-ring assembly in T3SS. *Mol. Microbiol.* **99**, 749–66 (2016).

20. Rocha, J. M. *et al.* Single-molecule tracking in live: Yersinia enterocolitica reveals distinct cytosolic complexes of injectisome subunits. *Integr. Biol. (United Kingdom)* **10**, 502–515 (2018).
21. Durisic, N., Laparra-Cuervo, L., Sandoval-Álvarez, Á., Borbely, J. S. & Lakadamyali, M. Single-molecule evaluation of fluorescent protein photoactivation efficiency using an in vivo nanotemplate. *Nat. Methods* **11**, 156–162 (2014).
22. Kusmierek, M. *et al.* A bacterial secreted translocator hijacks riboregulators to control type III secretion in response to host cell contact. *PLOS Pathog.* **15**, e1007813 (2019).
23. Wang, H. *et al.* Increased plasmid copy number is essential for Yersinia T3SS function and virulence. *Science (80-.).* **353**, 492–495 (2016).
24. Goulian, M. & Simon, S. M. Tracking Single Proteins within Cells. *Biophys. J.* **79**, 2188–2198 (2000).
25. Savin, T. & Doyle, P. S. Static and Dynamic Errors in Particle Tracking Microrheology. *Biophys. J.* **88**, 623–638 (2005).
26. Berglund, A. J. Statistics of camera-based single-particle tracking. *Phys. Rev. E* **82**, 011917 (2010).
27. Johnson, S. & Blocker, A. J. Characterization of soluble complexes of the Shigella flexneri type III secretion system ATPase. *FEMS Microbiol. Lett.* **286**, 274–8 (2008).
28. Lara-Tejero, M., Kato, J., Wagner, S., Liu, X. & Galán, J. E. A Sorting Platform Determines the Order of Protein Secretion in Bacterial Type III Systems. *Science (80-.).* **331**, 1188–91 (2011).
29. Prindle, J. R., Wang, Y., Rocha, J. M., Diepold, A. & Gahlmann, A. Distinct Cytosolic Complexes Containing the Type III Secretion System ATPase Resolved by Three-Dimensional Single-Molecule Tracking in Live Yersinia enterocolitica. *Microbiol. Spectr.* **10**, (2022).
30. Makino, F. *et al.* The Architecture of the Cytoplasmic Region of Type III Secretion Systems. *Sci. Rep.* **6**, 33341 (2016).
31. Hu, B. *et al.* Visualization of the type III secretion sorting platform of Shigella flexneri. *Proc. Natl. Acad. Sci.* **112**, 1047–1052 (2015).
32. Nans, A., Kudryashev, M., Saibil, H. R. & Hayward, R. D. Structure of a bacterial type III secretion system in contact with a host membrane in situ. *Nat. Commun.* **6**, 10114 (2015).
33. Wiley, D. J., Rosqvist, R. & Schesser, K. Induction of the Yersinia Type 3 Secretion System as an All-or-None Phenomenon. *J. Mol. Biol.* **373**, 27–37 (2007).
34. Enninga, J., Mounier, J. J., Sansonetti, P. J., Tran Van Nhieu, G. & Nhieu, G. T. Van. Secretion of type III effectors into host cells in real time. *Nat. Methods* **2**, 959–65 (2005).
35. Schlumberger, M. C. *et al.* Real-time imaging of type III secretion: Salmonella SipA injection into host cells. *Proc. Natl. Acad. Sci. U. S. A.* **102**, 12548–12553 (2005).
36. Eichelberg, K., Ginocchio, C. C. & Galán, J. E. Molecular and functional characterization of the Salmonella typhimurium invasion genes invB and invC: homology of InvC to the F0F1 ATPase family of proteins. *J. Bacteriol.* **176**, 4501–10 (1994).
37. Pozidis, C. *et al.* Type III protein translocase: HrcN is a peripheral ATPase that is activated by oligomerization. *J. Biol. Chem.* **278**, 25816–24 (2003).
38. Majewski, D. D. *et al.* Cryo-EM structure of the homohexameric T3SS ATPase-central stalk complex reveals rotary ATPase-like asymmetry. *Nat. Commun.* **10**, 626 (2019).
39. Roblin, P., Dewitte, F., Villeret, V., Biondi, E. G. & Bompard, C. A Salmonella Type Three Secretion Effector/Chaperone Complex Adopts a Hexameric Ring-Like Structure. *J. Bacteriol.* **197**, 688–698 (2015).

40. Bai, F. *et al.* Assembly dynamics and the roles of FliI ATPase of the bacterial flagellar export apparatus. *Sci. Rep.* **4**, 6528 (2014).

41. Akeda, Y. & Galán, J. E. Chaperone release and unfolding of substrates in type III secretion. *Nature* **437**, 911–915 (2005).

42. Spaeth, K., Chen, Y.-S. & Valdivia, R. The Chlamydia type III secretion system C-ring engages a chaperone-effector protein complex. *PLoS Pathog.* **5**, e1000579 (2009).

43. Lara-Tejero, M. *et al.* Role of SpaO in the assembly of the sorting platform of a Salmonella type III secretion system. *PLOS Pathog.* **15**, e1007565 (2019).
