# Supplementary material for: Cytosolic sorting platform complexes shuttle type III secretion system effectors to the injectisome in Yersinia enterocolitica
Source: Nat Microbiol. 2024 Jan 3;9(1):185–99. doi: 10.1038/s41564-023-01545-1 (PMC10769875; doi:10.1038/s41564-023-01545-1)
Supplement: Supplementary file 2 — Reporting Summary [file 41564_2023_1545_MOESM2_ESM.pdf]

nature portfolio

Corresponding author(s): Andreas Diepold & Ulrike Endesfelder

Last updated by author(s): Oct 20, 2023

Reporting Summary

Nature Portfolio wishes to improve the reproducibility of the work that we publish. This form provides structure for consistency and transparency in reporting. For further information on Nature Portfolio policies, see our Editorial Policies and the Editorial Policy Checklist.

Statistics

For all statistical analyses, confirm that the following items are present in the figure legend, table legend, main text, or Methods section.

n/a	Confirmed	
☐	☒	The exact sample size (*n*) for each experimental group/condition, given as a discrete number and unit of measurement
☐	☒	A statement on whether measurements were taken from distinct samples or whether the same sample was measured repeatedly
☐	☒	The statistical test(s) used AND whether they are one- or two-sided<br>*Only common tests should be described solely by name; describe more complex techniques in the Methods section.*
☒	☐	A description of all covariates tested
☒	☐	A description of any assumptions or corrections, such as tests of normality and adjustment for multiple comparisons
☐	☒	A full description of the statistical parameters including central tendency (e.g. means) or other basic estimates (e.g. regression coefficient) AND variation (e.g. standard deviation) or associated estimates of uncertainty (e.g. confidence intervals)
☐	☒	For null hypothesis testing, the test statistic (e.g. *F*, *t*, *r*) with confidence intervals, effect sizes, degrees of freedom and *P* value noted<br>*Give P values as exact values whenever suitable.*
☒	☐	For Bayesian analysis, information on the choice of priors and Markov chain Monte Carlo settings
☒	☐	For hierarchical and complex designs, identification of the appropriate level for tests and full reporting of outcomes
☒	☐	Estimates of effect sizes (e.g. Cohen's *d*, Pearson's *r*), indicating how they were calculated

Our web collection on statistics for biologists contains articles on many of the points above.

Software and code

Policy information about availability of computer code

Data collection	sptPALM images were acquired on a Nikon Ti Eclipse microscope, using the Micro-Manager software version 1.4.23.
Data analysis	Rapidstorm software version 3.3.1 was used for single-molecule localization. ImageJ 1.51v./1.52p/1.53c based Fiji software package was used for cell segmentation. Custom-written tracking software Swift was used for tracking, visualizing and filtering sptPALM data (http://bit.ly/swifttracking; version 0.3.1, used in this manuscript, and all subsequent versions of the software can also be obtained upon request to the authors). Binomial distribution fit analysis was performed with Python version 3.8.10 using the SciPy optimize library. Plotting of data was performed using Origin 2019.<br>Protein data was analyzed with MaxQuant in standard settings (Tyanova et al, 2016). If further statistical analysis was required, the MaxQuant "proteinGroups.txt" file was further evaluated using SafeQuant 2.4 (Ahrné et al, 2016, https://github.com/georgiaAngelidou/SafeQuant.v2.3.5, current versions available upon request to the authors).<br>Standard spreadsheet calculations were performed using Microsoft Excel 2016.

For manuscripts utilizing custom algorithms or software that are central to the research but not yet described in published literature, software must be made available to editors and reviewers. We strongly encourage code deposition in a community repository (e.g. GitHub). See the Nature Portfolio guidelines for submitting code & software for further information.

Data

Policy information about availability of data

All manuscripts must include a data availability statement. This statement should provide the following information, where applicable:

- Accession codes, unique identifiers, or web links for publicly available datasets
- A description of any restrictions on data availability
- For clinical datasets or third party data, please ensure that the statement adheres to our policy

All relevant data are included in the paper and/or its Supplementary information files. Source data are provided with this paper. The mass spectrometry proteomics data have been deposited to the ProteomeXchange Consortium via the PRIDE 104 partner repository with the dataset identifier PXD044214 (see Suppl. Table 6 for assignment). Supplementary videos are accessible at https://doi.org/10.17617/3.HMABQ2

Human research participants

Policy information about studies involving human research participants and Sex and Gender in Research.

Reporting on sex and gender	Not applicable.
Population characteristics	N/A
Recruitment	N/A
Ethics oversight	N/A

Note that full information on the approval of the study protocol must also be provided in the manuscript.

Field-specific reporting

Please select the one below that is the best fit for your research. If you are not sure, read the appropriate sections before making your selection.

☒ Life sciences ☐ Behavioural & social sciences ☐ Ecological, evolutionary & environmental sciences

For a reference copy of the document with all sections, see nature.com/documents/nr-reporting-summary-flat.pdf

Life sciences study design

All studies must disclose on these points even when the disclosure is negative.

Sample size	No preliminary sample-size calculation was determined. Samples sizes were chosen to allow a clear test of the conclusions drawn from the presented data based on similar experiments, or as n=3 where no such previous information was available. As the variance across experiments was low in most case, no explicit power calculations were considered necessary.
Data exclusions	No data were excluded.
Replication	Experiments were performed in independent replicates as indicated in the respective figure legends, material and methods, and/or the Supplementary Information, specifically Suppl. Table 2.
Randomization	Random allocation with regard to covariate is not applicable to the experiments carried out; no allocation into experimental groups was performed.
Blinding	Blinding was not required as the data processing did not rely on subjective assignments.

Reporting for specific materials, systems and methods

We require information from authors about some types of materials, experimental systems and methods used in many studies. Here, indicate whether each material, system or method listed is relevant to your study. If you are not sure if a list item applies to your research, read the appropriate section before selecting a response.

Materials & experimental systems

n/a	Involved in the study
☐	☒ Antibodies
☒	☐ Eukaryotic cell lines
☒	☐ Palaeontology and archaeology
☒	☐ Animals and other organisms
☒	☐ Clinical data
☒	☐ Dual use research of concern

Methods

n/a	Involved in the study
☒	☐ ChIP-seq
☒	☐ Flow cytometry
☒	☐ MRI-based neuroimaging

Antibodies

Antibodies used	Primary antibodies: rabbit antibodies against mCherry (Biovision 5993, 1:2000) or against the Flag peptide (Rockland 600-401-383S, 1:5000)<br>Secondary antibodies: anti-rabbit antibodies conjugated to horseradish peroxidase (HRP) (Sigma A8275, 1:5000)
Validation	Validation statements and examples on manufacturers' sites: https://www.abcam.com/mcherry-antibody-ab286186.html, https://www.rockland.com/categories/primary-antibodies/antibody-for-the-detection-of-flag-conjugated-proteins-600-401-383/, https://www.sigmaaldrich.com/GB/en/product/sigma/a8275. Additional validation by controls used in respective experiments.
